# Persistent poverty disparities in incidence and outcomes among oral and pharynx cancer patients

**DOI:** 10.1007/s10552-024-01867-3

**Published:** 2024-03-23

**Authors:** Shama Karanth, Shilpi Mistry, Meghann Wheeler, Tomi Akinyemiju, Joel Divaker, Jae Jeong Yang, Hyung-Suk Yoon, Dejana Braithwaite

**Affiliations:** 1 Department of Surgery, University of Florida College of Medicine, Gainesville, FL USA; 2 University of Florida Health Cancer Center, 2004 Mowry Road, Gainesville, FL 32610 USA; 3 Department of Epidemiology, University of Florida College of Public Health and Health Professions, Gainesville, FL USA; 4 Department of Population Health Sciences, Duke University School of Medicine, Durham, NC USA

**Keywords:** Oral and pharynx cancer, Cancer disparities, Survival, Race

## Abstract

**Purpose:**

Disparities in oral cavity and pharyngeal cancer based on race/ethnicity and socioeconomic status have been reported, but the impact of living within areas that are persistently poor at the time of diagnosis and outcome is unknown. This study aimed to investigate whether the incidence, 5-year relative survival, stage at diagnosis, and mortality among patients with oral cavity and pharyngeal cancers varied by persistent poverty.

**Methods:**

Data were drawn from the SEER database (2006–2017) and included individuals diagnosed with oral cavity and pharyngeal cancers. Persistent poverty (at census tract) is defined as areas where ≥ 20% of the population has lived below the poverty level for ~ 30 years. Age-adjusted incidence and 5-year survival rates were calculated. Multivariable logistic regression was used to estimate the association between persistent poverty and advanced stage cancer. Cumulative incidence and multivariable subdistribution hazard models were used to evaluate mortality risk. In addition, results were stratified by cancer primary site, sex, race/ethnicity, and rurality.

**Results:**

Of the 90,631 patients included in the analysis (61.7% < 65 years old, 71.6% males), 8.8% lived in persistent poverty. Compared to non-persistent poverty, patients in persistent poverty had higher incidence and lower 5-year survival rates. Throughout 10 years, the cumulative incidence of cancer death was greater in patients from persistent poverty and were more likely to present with advanced-stage cancer and higher mortality risk. In the stratified analysis by primary site, patients in persistent poverty with oropharyngeal, oral cavity, and nasopharyngeal cancers had an increased risk of mortality compared to the patients in non-persistent poverty.

**Conclusion:**

This study found an association between oral cavity and pharyngeal cancer outcomes among patients in persistent poverty indicating a multidimensional strategy to improve survival.

**Supplementary Information:**

The online version contains supplementary material available at 10.1007/s10552-024-01867-3.

## Introduction

In 2023, an estimated 54,540 individuals are projected to  be diagnosed with oral cavity and pharyngeal cancers in the United States, accounting for 2.8% of all newly diagnosed cancer cases [1]. Despite an overall 5-year survival rate of ~ 68%, persistent disparities exist that adversely affect survival rates among disadvantaged groups, specifically individuals belonging to lower socioeconomic status (SES) [2–4]. Among the various measures of SES, neighborhood poverty or regional deprivation has been recognized as an important predictor of unfavorable oral cavity and pharyngeal cancer outcomes [2, 3]. Within this context, the concept of persistent poverty has gained attention in the field of cancer disparities research as a more appropriate measure of poverty compared to the assessment of the current level of poverty [5, 6].

Persistent poverty refers to areas in which a minimum of 20% of the population has lived below the poverty level for approximately 30 years [5–7]. These areas exhibit substantial distinctions from other regions, often having higher concentrations of racial/ethnic minorities [8], larger numbers of children [5], elevated rates of unemployment [5, 9], and are often located in rural communities [8–10]. Moreover, individuals residing in areas marked by persistent poverty face an increased risk of developing cancer due to an increased susceptibility to cancer risk factors, as well as a greater probability of cancer-related mortality when compared to those inhabiting regions characterized by current, yet non-persistent poverty [6, 7, 11]. Some of the risk factors for the disparities between persistent poverty and non-persistent poverty include reduced access to healthcare services [12], lower utilization of cancer screening [13, 14], and an increase in cancer risk behaviors such as smoking, alcohol consumption, and human papillomavirus infections [10]. In addition, poverty and other factors of SES (education, income, lack of insurance, unemployment) that are prevalent in poverty-stricken areas are crucial determinants of cancer outcomes [8]. Individuals living in poverty often lack access to fundamental necessities, such as stable housing, nutritious food, safe environments, and high-quality healthcare services, and importantly influence receipt of guideline-adherent treatment, as well as active surveillance following treatment [10, 12].

To date, the relationship between persistent poverty and the incidence rates, stage at diagnosis, and outcomes of oral cavity and pharyngeal cancer have not been thoroughly evaluated. Therefore, the purpose of this study is to examine the association between the incidence and outcomes of oral cavity and pharyngeal cancer and persistent poverty. This study holds the potential to offer significant insights into the complex interplay between poverty and oral cavity and pharyngeal cancers, thereby informing policies and interventions aimed at enhancing cancer prevention, screening, and treatment in disadvantaged communities.

## Materials and methods

### Data source and study population

We utilized data from the Surveillance, Epidemiology, and End Results (SEER) database (RRID: SCR_006902) maintained by the National Cancer Institute (NCI) using SEER*Stat Software version 8.4.0 (http://seer.cancer.gov/seerstat/) and acquired the specialized SEER Research Plus Data (Specialized with Census Tract SES/Rurality), 18 Registries (excl AK), Nov 2020 Sub (2006–2018), which covers ∼28% of the U.S. population [15]. We included individuals with a primary diagnosis of oral cavity or salivary gland and pharynx (including hypopharynx, nasopharynx, and oropharynx) cancers between 2006 and 2017. A total of 101,804 oral cavity and pharyngeal cancer cases were defined using the International Classification of Diseases for Oncology, 3rd Edition (ICD-O-3) site classification system [16]. We then excluded individuals diagnosed at autopsy/ death certificates (*n* = 10,791), individuals with missing data for rurality, or individuals who were American Indian/Alaska Natives (*n* = 377), leaving a total sample size of 90,631 patients (Supplementary Table 1). This work was deemed exempt from IRB review as all data utilized are publicly available. Oral cavity and pharyngeal cancers was defined by the primary sites and categorized into the oral cavity—oral and salivary glands (codes C00.0-C00.9, C02.0-C02.3, C02.8, C02.9, C03.0, C03.1, C03.9, and C04.0, C04.1, C04.8, C04.9, C05.0, C06.0-C06.2, C06.8, C06.9, C07.8, C07.9, C08.0, C08.1, C08.8, C08.9], oropharynx (codes C05.1, C05.2, C05.8, C05.9C01.9, C02.4, C09.0, C09.1, C09.8, C09.9, C10.0-C10.4, C10.8, C10.9, C14.0, C14.2, C14.8), hypopharynx (C12.9, C13.0-C13.2, C13.8, C13.9), and nasopharynx (C11.0-C11.3, C11.8, C11.9) [16].

#### Persistent poverty

The persistent poverty variable identifies census tracts as being persistently poor if 20% or more of the population has lived below the poverty level for a period spanning about 30 years based on the 1990, 2000 decennial censuses, and 2007–2011 and 2015–2019 American Community Survey 5-year estimates. It was developed by the NCI in collaboration with the US Department of Agriculture, Economic Research Service (ERS) [5, 7, 17]. Persistent poverty was classified as persistent poverty or non-persistent poverty (reference group).

### Outcomes

#### Advanced stage

The stage at diagnosis was defined using the SEER Summary Stage variable, which was categorized into Localized, Regional, Distant, and Unknown/unstaged. The stage at diagnosis was then aggregated into either advanced stage (regional/distant) or early stage (localized). Unknown/unstaged cancers were not included in the regression analysis.

#### Mortality

All-cause mortality was defined as the time from diagnosis until death from any cause [18], while cancer-specific mortality was defined as the time from diagnosis until death from cancer [19]. All mortality rates were calculated as the number of deaths/1,000 person-years.

#### Covariates

The SEER database was used to acquire demographic and clinical characteristics [20]. Race/ethnicity was classified as Non-Hispanic (NH) White, NH-Black, NH-Asian Pacific Islander (Asian/PI), and Hispanic [20]. From this point forward, racial/ethnic groups will be referred to without the NH prefix. Patient-level demographics included age at diagnosis (20–49, 50–64, 65–79, and 80 years and above), sex (female vs. male), and marital status (married/partnered vs. never married/divorced/separated/widowed/unknown).

Tumor-related characteristics included treatments received, which comprised of surgery (yes/no), radiation (yes/no), and chemotherapy (yes/no/unknown), as well as the year of diagnosis (2006–2012 vs. 2013–2017). To classify the urban or rural patients, we used the 2013 Rural–Urban Continuum codes that distinguish metropolitan counties by the population size of their metro area, and nonmetropolitan counties by the degree of urbanization and adjacency to a metro area. A county was defined as rural if it was not within a metropolitan statistical area and had an urban population of less than 20,000 [21].

### Statistical analysis

We first generated descriptive statistics to characterize distributions in the overall study sample as well as by persistent poverty status. Characteristics of participants by persistent poverty status were assessed with analysis of variance or χ2 tests. Age-adjusted incidence rates (and their standard errors) per 100,000 and 5-year relative survival rates were calculated for combined oral cavity and pharyngeal cancers as well as by specific cancer site using SEER*Stat. Incidence rate ratios (IRRs) and 95% confidence intervals (95% CIs) were calculated using the Tiwari method [22] and age adjusted to the U.S. standard population (aged ≥ 20 years) by persistent poverty status and by primary cancer site.

We summarized the number of deaths, person-years during follow-up, and mortality rate/1,000 person for oral cavity and pharyngeal cancer survivors. Adjusted cumulative incidence function curves with 95% CIs were generated to describe the incidence over time of death for oral cavity and pharyngeal cancer-specific death by persistent poverty status and further stratified by primary site [23]. Multivariable logistic regression models were run to evaluate the odds of being diagnosed with advanced-stage oral cavity and pharyngeal cancer by persistent poverty adjusted for age, sex, race/ethnicity, marital status, rurality, and year of diagnosis. In addition, subgroup analyses were conducted for sex, rurality, and race/ethnicity to explore their interaction with persistent poverty. In each subgroup analysis, we created an interaction term between persistent poverty and the variable used for stratification; the Wald test was used to assess if the interaction was significant, with a *p* value < 0.05 suggesting statistical heterogeneity between subgroups. Adjusted hazard ratios (aHR) and 95% CIs were estimated using Cox proportional hazards regression models to predict the risk of all-cause mortality. The Cox proportional hazards assumption was examined using the Schoenfeld residual method [24]. Adjusted multivariable competing risk hazard models (Fine and Gray's subdistribution) were used to estimate subdistribution HR (sHR) with a 95% CI for cancer-specific mortality by persistent poverty status. Patients who died from other non-cancer causes were considered competing risks. Models were adjusted for sex, age, race/ethnicity, marital status, stage, rurality, primary site, treatment variables (surgery, radiotherapy, chemotherapy), histology, and year of diagnosis. Cancer site-specific hazard ratios were additionally adjusted for the same covariates except for the primary site. Patients who died from other non-oral cavity and pharyngeal cancer causes were considered to have died from competing risks. Subgroup analyses were used to evaluate potential effect modification by sex, race/ethnicity, or rurality, with the same adjustments as the full model except for the stratified variable. An interaction term between these variables and poverty was generated and added to the model; a Wald test was used to assess whether the interaction was significant. The proportion of missing covariates was < 10%, thus we did not use imputation to handle that in the analysis. The data were analyzed using SEER*Stat and SAS version 9.4. Statistical significance was set at *p* value < 0.05 and the Holm Bonferroni procedure was used to preserve the family-wise type-1 error rate for multiple comparisons within each set of analyses [25].

## Results

A total of 90,631 individuals with oral cavity and pharyngeal cancers were included in the study, with 7,985 (8.8%) residing in persistent poverty areas. Most patients were between 50 and 64 years old (45.4%), male (71.6%), White (75.2%), diagnosed with regional stage cancer (48.6%), and resided in urban counties (87.6%) (Table 1). The most common oral cavity and pharyngeal cancer sites were the oropharynx (44.9%) and oral cavity (44.2%).

**Table 1:** Patient characteristics of oral cavity and pharynx cases by persistent poverty status

Characteristic *N* (%)	Overall *N* = 90,631	Persistent poverty *N* = 7985	Non-persistent poverty *N* = 82,646	*p* value
*Age groups, year*				< .0001
20–49 years	14,733 (16.3)	1,419 (17.8)	13,314 (16.1)	
50–64 years	41,162 (45.4)	3,914 (49.0)	37,248 (45.1)	
65–79 years	26,101 (28.8)	2,086 (26.1)	24,016 (29.1)	
80 + years	8,635 (9.5)	567 (6.3)	8,068 (9.7)	
*Sex*				0.0840
Male	64,929 (71.6)	5,787 (72.5)	59,142 (71.6)	
Female	25,702 (28.4)	2,198 (27.5)	23,504 (29.4)	
*Race/ethnicity*				< .0001
NH-White	68,114 (75.2)	3,770 (47.2)	64,344 (77.9)	
NH-Black	8,187 (9.0)	2,739 (34.3)	5,448 (6.6)	
NH-Asian/PI	6,872 (7.6)	380 (4.8)	6,362 (7.8)	
Hispanic	7,458 (8.2)	1,096 (13.7)	6,492 (7.7)	
*Marital status*				< .0001
Married or partnered	47,797 (52.7)	2,727 (34.2)	45,070 (54.5)	
Other	42,834 (47.3)	5,258 (65.9)	37,576 (45.5)	
*Advanced stage*				< .0001
Localized	26,377 (29.1)	1,855 (23.2)	24,522 (29.7)	
Regional	44,066 (48.6)	3,797 (47.6)	40,269 (48.7)	
Distant	16,291 (18.0)	1,996 (25.0)	14,295 (17.3)	
Unknown/unstaged	3,897 (4.3)	337 (4.2)	3,560 (4.3)	
*Primary site*				< .0001
Oral	40,100 (44.2)	3,246 (40.7)	36,854 (44.6)	
Oropharynx	40,682 (44.9)	3,598 (45.1)	37,084 (44.8)	
Hypopharynx	4,426 (4.9)	629 (7.8)	3,797 (4.6)	
Nasopharynx	5,423 (6.0)	512 (6.4)	4,911 (6.0)	
*Surgery*				< .0001
Yes	47,783 (52.7)	3,423 (42.9)	44,360 (53.7)	
No	42,848 (47.3)	4,562 (57.1)	38,286 (46.3)	
*Radiotherapy*				0.0393
Yes	57,435 (63.4)	5,145 (64.4)	52,290 (63.3)	
No	33,196 (36.6)	2,840 (35.6)	30,356 (36.7)	
*Chemotherapy*				< .0001
Yes	42,086 (46.4)	3,935 (49.3)	38,151 (46.2)	
No/Unknown	48,545 (53.6)	4,050 (50.7)	44,495 (53.8)	
*Year of Diagnosis*				< .0001
2006–2012	49,606 (54.7)	4,581 (57.4)	45,025 (54.5)	
2013–2017	41,025 (45.3)	3,404 (42.6)	37,621 (45.5)	
*Rurality*				< .0001
Urban	79,348 (87.6)	5,978 (74.9)	73,370 (88.8)	
Rural	11,283 (12.5)	2,007 (25.1)	9,276 (11.2)	
*Years since diagnosis median (IQR)*	3.3 (1.4–6.7)	2.3 (0.8–5.3)	3.5 (1.4–6.8)	< .0001

*NH* non-Hispanic, *AJCC* American Joint Committee on Cancer, *IQR* interquartile range, *Oral* Oral cavity and salivary glands

Patients residing in areas with persistent poverty were more likely to reside in rural areas (persistent poverty [PP]: 25.1% vs non-persistent poverty [NPP]: 11.2%), were less likely to receive surgical resection (PP: 42.9% vs NPP: 53.7%), were less likely to be married or partnered (PP: 34.2% vs NPP: 54.5%), and were more likely to be Black (PP: 34.3% vs NPP: 6.6%), The age-adjusted incidence rate of oral cavity and pharyngeal cancer was significantly higher in persistent poverty areas compared to the non-persistent poverty areas, with rates of 18.7 (95% CI 18.1, 19.2) per 100,000 and 16.3 (95% CI 16.2, 16.4) per 100,000, respectively (Table 2). Persistent poverty census tracts showed higher site-specific incidence rates of cancer for all primary sites compared to non-persistent poverty census tracts. Both overall and site-specific incidence rate ratios (IRR) showed higher incidence in persistent poverty areas (oral cavity and pharyngeal cancer IRR: 1.14, 95% CI 1.11, 1.17; Oral cavity IRR: 1.05, 95% CI 1.01, 1.09; Oropharynx IRR: 1.28, 95% CI 1.21, 1.36; Nasopharynx IRR: 1.50, 95% CI 1.28, 1.75, Hypopharynx IRR: 1.49, 95% CI 1.32, 1.68) (Supplementary Table 1). In addition, incidence rates by sex, race/ethnicity, and rurality found significant differences in incidence rates between persistent poverty areas and non-persistent poverty areas across most demographics (Supplementary Table 2). Interestingly, an inverse relationship was seen among Hispanic patients, as patients in non-persistent poverty areas had a higher incidence of oral cavity and pharyngeal cancer. The overall five-year relative survival rate for oral cavity and pharyngeal cancer was lower in persistent poverty (48.2%, 95% CI 46.4, 49.4) compared to non-persistent poverty census tracts (67.2%, 95% CI 66.8, 67.6) (Table 2). Similarly, site-specific survival rates showed a lower survival rate in persistent poverty census tracts for all cancer sites. However, 5-year relative survival between persistent poverty areas and non-persistent poverty areas by sex, race/ethnicity, and rurality, no significant differences were observed.

**Table 2:** Oral cavity and pharynx cancer incidence rates and five-year relative survival rates by persistent poverty status

	Persistent poverty	Non-persistent poverty
Age-adjusted Incidence rates (per 100,000)^a^		
Oral and Pharynx	18.7 (18.1, 19.2)#	16.3 (16.2, 16.4)
*Site-specific*		
Oral	11.7 (11.2, 12.1)#	11.1 (11.0, 11.2)
Oropharynx	5.3 (5.0, 5.6)#	4.1(4.1, 4.2)
Nasopharynx	0.8 (0.6, 0.9)#	0.5 (0.5, 0.5)
Hypopharynx	1.2 (1.1, 1.3)#	0.8 (0.8, 0.8)
Five-year relative survival rates (%, 95% CI)		
Oral and Pharynx	48.2 (46.4, 49.4)	67.2 (66.8, 67.6)
*Site-specific*		
Oral	52.3 (50.6, 54.0)	68.6 (68.1, 69.1)
Oropharynx	46.2 (43.8, 48.6)	70.5 (69.8, 71.3)
Nasopharynx	47.4 (42.5, 52.1)*	61.6 (60.1,63.2)
Hypopharynx	23.9 (20.2,27.8)	37.0 (35.2, 38.7)

^a^Incidence rates are per 100,000 and age-adjusted to the 2000 US standard population

*The relative cumulative survival increased from a prior interval

^#^*p* value < 0.05, indicates that the rate ratio is significantly different than the rate for the Non-persistent poverty census tract

**Fig. 1:**
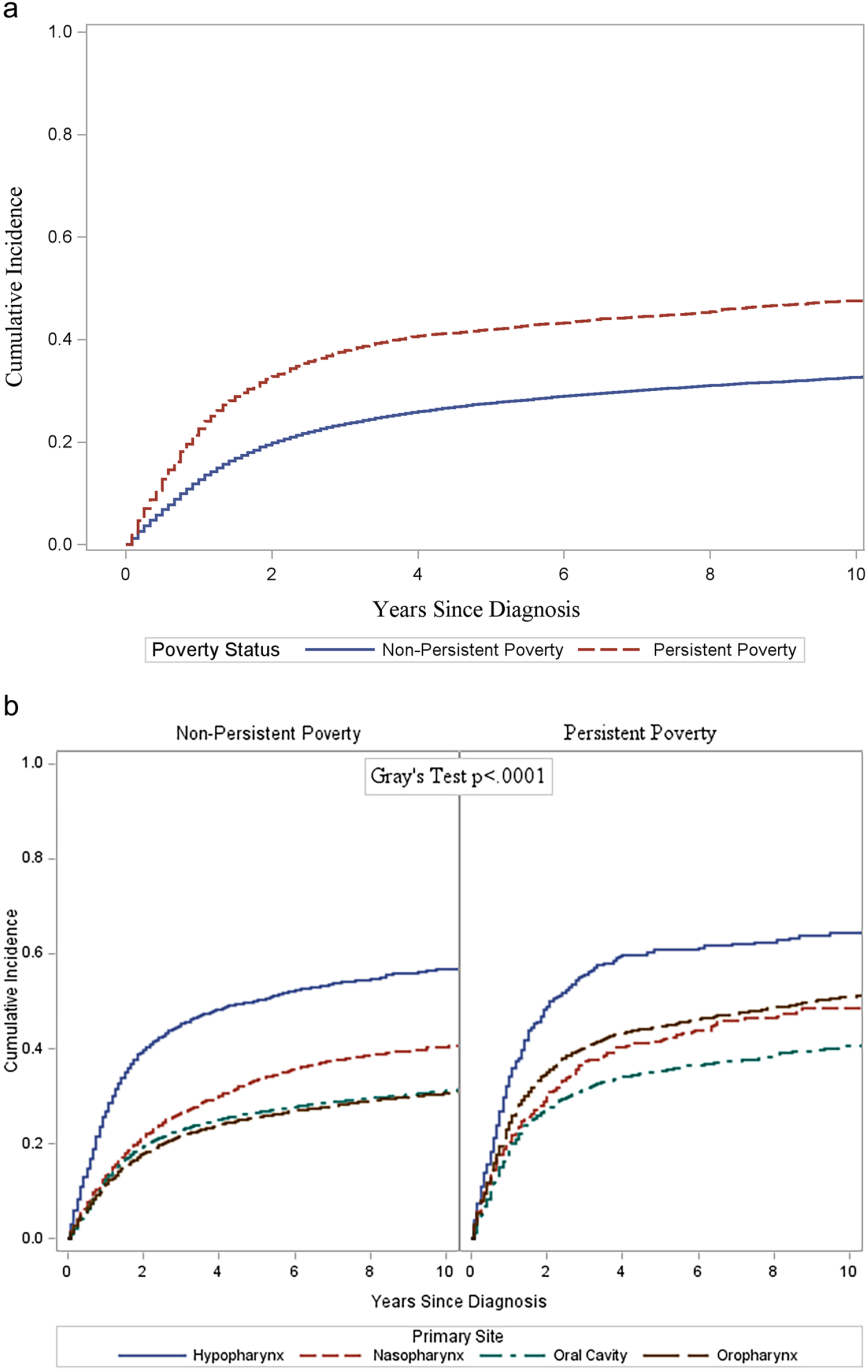
**a** Cumulative incidence of cancer-specific death and oral and pharynx cancer by persistent poverty status. **b** Cumulative incidence of cancer-specific death by persistent poverty status and primary site. Oral cavity includes oral and salivary gland cancers

The cumulative incidence of cancer-specific death was consistently higher among individuals residing in persistent poverty areas compared to those residing in non-persistent poverty areas (*p* < 0.0001) (Fig. 1a). The cumulative incidence of cancer-specific death was also consistently higher in areas with persistent poverty versus those without, stratified by primary cancer site (Fig. 1b). The curves illustrate that in both persistent poverty areas and non-persistent poverty areas, cancer of the hypopharynx had the highest incidence of cancer-specific death. The most notable difference in cancer-specific death by persistent poverty status is seen in oropharynx cancer, with persistent poverty displaying nearly double the cumulative incidence rate of areas in non-persistent poverty.

In our multivariable logistic regression analysis, the odds of being diagnosed with advanced-stage oral cavity and pharyngeal cancer (Table 3) we found that individuals residing in persistent poverty areas had a higher risk of being diagnosed with advanced-stage disease (OR 1.24, 95% CI 1.16, 1.32). Subgroup analysis by sex revealed similar increased odds among both females (OR 1.31, 95% CI 1.17, 1.46) and males (OR 1.21, 95% CI 1.12, 1.32). In the subgroup analysis by rurality, persistent poverty did not affect the odds of being diagnosed with advanced-stage oral cavity and pharyngeal cancer among individuals in rural areas but was associated with increased odds of advanced-stage disease in urban areas (OR 1.29, 95% CI 1.19, 1.39). Finally, in our analysis by race/ethnicity, living in a persistent poverty area was associated with increased odds of advanced-stage disease among White (OR 1.20, 95% CI 1.10, 1.31), Black (OR 1.25, 95% CI 1.10, 1.44), and Hispanic (OR 1.27, 95% CI 1.08, 1.49) individuals, while no association was observed for Asian/PI race. However, no significant interactions were identified among these strata.

**Table 3:** Adjusted models for odds of advanced stage (Regional/Distant vs Localized) at diagnosis (*n* = 86,734)

	Regional/distant stage *n/N* (%)	OR (95% CI)
Non-persistent poverty	54,564/79086 (66.0)	Ref.
Persistent poverty	5793/7648 (72.6)	1.24 (1.16, 1.32)
*Female*		
Non-persistent poverty	12,372/23504 (52.6)	Ref.
Persistent poverty	1392/2198 (63.3)	1.31 (1.17, 1.46)
*Male*		
Non-persistent poverty	42,192/59142 (71.3)	Ref.
Persistent poverty	4401/5787 (76.1)	1.21 (1.12, 1.32)
		*P*-interaction = 0.342
*Rural*		
Non-persistent poverty	5774/9276 (62.3)	Ref.
Persistent poverty	1359/2007 (67.7)	1.10 (0.97 1.25)
*Urban*		
Non-persistent poverty	48,790/73370 (66.5)	Ref.
Persistent poverty	4434/5978 (74.2)	1.29 (1.19, 1.39)
		*P-*interaction = 0.097
*Race*		
*NH-White*		
Non-persistent poverty	42,075/64344 (65.4)	Ref.
Persistent poverty	2582/3770 (68.5)	1.20 (1.10 1.31)
*NH-Black*		
Non-persistent poverty	4074/5448 (74.9)	Ref.
Persistent poverty	2211/2739 (80.7)	1.25 (1.10, 1.44)
*Hispanic*		
Non-persistent poverty	4145/6362 (65.2)	Ref.
Persistent poverty	735/1096 (67.1)	1.27 (1.08, 1.49)
*Asian/PI*		
Non-persistent poverty	4270/6492 (65.8)	Ref.
Persistent poverty	95/380 (69.7)	1.07 (0.85, 1.35)
		*P*-interaction = 0.275

Advanced stage includes regional and distant stages based on SEER summary stage. Models are adjusted for age, sex, primary site, marital status, rurality, year of diagnosis, and histology. The models in the subgroup analyses are adjusted for the same set of covariates as the primary multivariable model except the variable used for stratification

*n* number of advanced stage, *N* number of individuals, *NH* non-Hispanic, *PI* Pacific Islander, *OR* odds ratio, *CI* confidence interval.

In the Cox proportional hazard models for all-cause and cancer-specific mortality (Table 4), compared to patients residing in areas with non-persistent poverty, patients residing in areas with persistent poverty had a higher risk of all-cause mortality (aHR: 1.31, 95% CI 1.26, 1.35) and cancer-specific mortality (sHR: 1.28, 95% CI 1.23, 1.33). When stratified by cancer site, patients residing in areas in persistent poverty diagnosed with oropharynx cancer had a higher risk of all-cause mortality (aHR: 1.46, 95% CI 1.38, 1.53) and cancer-specific mortality (sHR: 1.47, 95% CI 1.38, 1.56). Higher risk of all-cause mortality only was found among patients residing in areas with persistent poverty diagnosed with oral cavity cancer (aHR: 1.13, 95% CI 1.07, 1.19) and nasopharynx cancer (aHR: 1.30, 95% CI 1.15, 1.49). Although there was no difference in risk of mortality in hypopharyngeal cancers, the death rate was higher among residents in persistent poverty (241.2 vs. 178.4/1,000 deaths) (Table 4).

**Table 4:** Cox-proportional hazards’ models predicting risk of all-cause and cancer-specific mortality for persistent poverty status

	All-cause mortality		Cause-specific mortality	
	No. of deaths /1000 person-years (95% CI)	aHR (95% CI)	No. of deaths /1000 person-years (95% CI)	sHR (95% CI)
Oral and Pharynx	98.6 (97.6, 99.6)	1.31 (1.26, 1.35)	67.0 (66.2, 67.8)	1.28 (1.23, 1.33)
*Site-specific*				
Oral Cavity	98.3 (96.8, 99.8)	1.13 (1.07, 1.19)	62.3 (61.2, 63.5)	1.10 (1.02, 1.18)
Oropharynx	88.2 (86.8, 89.5)	1.46 (1.38, 1.53)	62.8 (60.7, 63.0)	1.47 (1.38, 1.56)
Nasopharynx	101.4 (97.4, 105.5)	1.30 (1.15, 1.49)	79.1 (75.5, 82.7)	1.14 (0.96, 1.34)
Hypopharynx	241.2 (232.9, 249.8)	1.14 (1.00, 1.24)	178.4 (171.2, 185.8)	1.05 (0.92, 1.20)

Model 1: aHRs were adjusted for sex, age, race/ethnicity, marital status, stage, rurality, primary site, treatment, histology, and year of diagnosis. Site-specific aHRs were adjusted for the same covariates except for the primary site

*NH* non-Hispanic, *aHR* adjusted hazard ratio, *sHR* subdistribution hazard ratio, *Oral cavity* oral cavity and salivary gland

In addition, when stratified by sex, males residing in persistent poverty areas had both a higher risk of all-cause mortality (aHR: 1.34, 95% CI 1.29, 1.39) and cancer-specific mortality (sHR: 1.31, 95% CI 1.24, 1.37) compared with females (Table 5). When stratified by race/ethnicity, significant excess all-cause and cancer-specific mortality risk were seen among all races/ethnicities, with the highest excess among White patients and Asian/PI patients. Finally, by rurality, patients residing in urban areas with persistent poverty had a higher risk of all-cause mortality (aHR: 1.33, 95% CI 1.28, 1.38) and a higher risk of cancer-specific mortality (sHR: 1.31, 95% CI 1.25, 1.36) when compared to patients residing in rural areas with persistent poverty.

**Table 5:** Cox-proportional hazards’ models predicting risk of all-cause and cancer-specific mortality for persistent poverty status by sex, race/ethnicity and rurality

	All-cause mortality		Cancer-specific mortality	
	No. of deaths /1,000 person-years (95% CI)	aHR (95% CI)	No. of deaths /1,000 person-years (95% CI)	aHR (95% CI)
*Sex*				
Male	100.1 (99.0, 101.3)	1.34 (1.29, 1.39)	68.3 (67.3, 69.3)	1.31 (1.24, 1.37)
Female	94.7 (92.9, 96.5)	1.19 (1.11, 1.27)	63.7 (62.3, 65.2)	1.19 (1.11, 1.27)
*p interaction*		*p* value = 0.0002		*p* value = 0.0002
*Race/Ethnicity*				
NH-White	93.8 (92.8, 95.0)	1.41 (1.34, 1.47)	61.3 (60.4, 62.2)	1.35 (1.27, 1.43)
NH-Black	163.1 (158.5, 167.8)	1.20 (1.14, 1.28)	122.0 (118.0, 126.1)	1.20 (1.12, 1.29)
NH-Asian/PI	81.8 (78.6, 85.1)	1.44 (1.24, 1.67)	63.2 (60.4, 66.1)	1.36 (1.14, 1.62)
Hispanic	101.3 (97.7, 104.9)	1.21 (1.10, 1.32)	75.7 (72.7, 78.9)	1.27 (1.13, 1.42)
*p interaction*		*p* value < .0001		*p* value = 0.069
*Rurality*				
Urban	96.7 (95.6, 97.7)	1.33 (1.28, 1.38)	66.0 (65.1, 66.8)	1.31 (1.25, 1.36)
Rural	112.5 (109.5, 115.6)	1.23 (1.15, 1.32)	74.5 (72.0, 77.0)	1.16 (1.07, 1.28)
*p interaction*		*p* value < .0001		*p* value = 0.0011

The models are adjusted for sex, age, marital status, stage, rurality, primary site, treatment, histology, and year of diagnosis, except for the stratified variable in the models

*NH* non-Hispanic, *aHR* adjusted hazard ratio

## Discussion

This population-based study investigated the role of census-tract-level persistent poverty on oral cavity and pharyngeal cancer incidence, stage at diagnosis, and survival. Individuals from persistent poverty areas had higher oral cavity and pharyngeal cancer incidence rates, and worse outcomes including higher death rates, higher odds of advanced disease at diagnosis, and higher rates of all-cause and cancer-specific mortality. The findings highlight the inequities in oral cavity and pharyngeal cancer outcomes among patients living in persistent poverty.

To our knowledge, this is the first study to characterize the association between persistent poverty and oral cavity and pharyngeal cancer incidence, 5-year relative survival rates, stage at diagnosis, and mortality in the U.S. By definition, persistent poverty is different from chronic poverty, persistent poverty relates to geographic locations with high poverty rates for an extended time, unlike chronic poverty which identifies individuals/families in poverty over time [26]. In this study, patients residing in areas with persistent poverty were more likely to be Black, from rural counties, and less likely to receive surgical resection compared to their counterparts in non-persistent poverty. Prior research studies have identified differences by race, SES, insurance status, income, distance to care, hospital volume, and facility type [4, 27–32], this study adds persistent poverty to the list of factors that affect the diagnosis and outcomes of oral cavity and pharyngeal cancer. These results are consistent with Moss et al. previous work that examined the association of persistent poverty and all cancer sites and additionally by specific cancer sites with high mortality burden. The authors report absolute and/or relative disparities in mortality by persistent poverty that were widened particularly for four cancer outcomes namely lung and bronchus; colorectal; liver and intrahepatic bile duct; and breast [7]. The authors additionally report the interacting role of persistent poverty in two study periods 1990–1992 vs. 2014–2018 and observed the highest mortality rates among Black rural residents [7].

In the current study, we found higher incidence rates of oral cavity and pharyngeal cancer, which aligns with prior research that has demonstrated risk factors such as higher tobacco and alcohol use, poor nutrition, other factors such as the non-availability of specialists for oral cancer screening, and lower uptake of HPV vaccines are prevalent in impoverished residential areas [33]. This warrants public health interventions such as education on improving lifestyle and behavioral risk factors and healthcare resources to facilitate access to preventive treatment such as oral cancer screening. Thus, disparities in preventive care may cause individuals from persistent poverty areas to present with advanced-stage oral cavity and pharyngeal cancer resulting in poor survival outcomes. In a population-based study, Kravietz et al. found individuals from lower SES groups and racial minorities were less likely to receive head and neck cancer screening examinations in which a doctor or dentist pulls on the tongue and conducts a neck exam [34]. Furthermore, the authors also report that the majority of participants who have smoked > 100 cigarettes had never received adequate screening examination for head and neck cancer nor had they received education on smoking cessation and screening for cancer [34]. Hence additional research on other factors such as access to health care, and potential synergistic biological mechanisms driving these disparities is needed.

Our study underscores a significant disparity in cancer-specific mortality between persistent and non-persistent poverty areas, with the most prominent divergence observed in the oropharynx, wherein patients residing in the persistent poverty areas exhibited nearly twice the cumulative incidence of cancer-specific mortality compared to non-persistent poverty areas. However, compared to the other oral cavity and pharyngeal cancer sites, a higher cumulative incidence of hypopharynx cancer-specific death was seen among residents from both persistent poverty and non-persistent poverty areas. Hypopharyngeal cancer survival rates have not improved over the years, mainly due to detection at late stages and asymptomatic progression [35, 36]. Therefore, our study provides evidence that additional research is warranted to improve survival for this cancer.

Historical studies have demonstrated a clear correlation between material disadvantage (particularly residing in poverty-stricken areas) and an elevated risk of developing chronic illnesses, including cancer [6, 37, 38]. This connection can be attributed to a multitude of factors [8], including persistent stress stemming from numerous risk factors prevalent in areas identified as persistent poverty [39]. The enduring burden of stress can induce physiological abnormalities, such as chronic inflammation, thereby amplifying the susceptibility to cancer among individuals residing in economically disadvantaged communities [37]. Recognizing the impact of persistent poverty on the development of oral cavity and pharyngeal cancers is important as it lays the foundation for the formulation of effective strategies aimed at mitigating health disparities and enhancing outcomes for these high-risk populations. By accounting for the multifaceted associations between poverty and oropharyngeal cancer, tailored interventions and targeted approaches can be devised to ameliorate the unequal burden faced by vulnerable populations.

## Strengths and limitations

The strengths of this study included the large population-based sample from the SEER database which allowed for stratification by cancer site subtype, and the use of SEER data ensured that all variables were standardized. Our study evaluated various effect modifiers, such as sex, race/ethnicity, and rurality, to investigate their association with all-cause and cancer-specific mortality. However, one of the limitations of the study is that it only considered the impact of persistent poverty at the census tract level, and it may not reflect the true socioeconomic status of individuals. Further, we were unable to account for HPV status at time of diagnosis as this was missing among most patients in the final sample. Finally, due to database limitations, we were unable to account for other important factors that may influence cancer outcomes, such as insurance, education, lifestyle factors, and comorbidities.

In conclusion, patients diagnosed with oral and pharyngeal cancers residing in areas characterized by persistent poverty were found to have a higher incidence, lower 5-year survival rates, greater prevalence of advanced-stage disease, and elevated risk of mortality compared to patients residing in areas without persistent poverty. Consequently, reducing the burden, achieving favorable cancer outcomes, and mitigating disparities require targeted, multilevel interventions [6] aimed at improving access to care and reducing cancer treatment/outcome inequalities for patients living in persistent poverty regions. Along with poverty reduction strategies, improving the health of individuals should be an integral component of cancer prevention, screening, and treatment initiatives in disadvantaged communities.

### Supplementary Information

Below is the link to the electronic supplementary material.

Supplementary file1 (DOCX 37 KB)

## Data Availability

The data studied in this investigation are accessible from the NCI's SEER database (https://seer.cancer.gov/data-software/).
