# Supplementary material for: Persistent poverty disparities in incidence and outcomes among oral and pharynx cancer patients
Source: Cancer Causes Control. 2024 Mar 23;35(7):1063–73. doi: 10.1007/s10552-024-01867-3 (PMC11217118; doi:10.1007/s10552-024-01867-3)
Supplement: Supplementary file 1 — Supplementary file1 (DOCX 37 KB) [file 10552_2024_1867_MOESM1_ESM.docx]

Supplementary Figure 1. Flowchart of study participants selection, SEER 2006-2017

SEER (2006-2017)

N= 101,804

Excluded n= 10,791

Diagnosed at autopsy only, death certificate only

Participants

n= 91,008

Excluded =377

Participants with missing rurality

American Indian/Alaska Native

Participants

n= 90,631

Supplementary Table 1: Incidence Rate Ratios by Persistent Poverty

Rate Ratios comparing Persistent vs Non-Persistent poverty

|  | Incidence Rate Ratios (95%CI) |
| --- | --- |
| Oral and Pharynx | 1.14 (1.11, 1.17) |
| ***Site-specific*** |  |
| Oral Cavity | 1.05 (1.01, 1.09) |
| Oropharynx | 1.28 (1.21, 1.36) |
| Nasopharynx | 1.50 (1.28, 1.75) |
| Hypopharynx | 1.49 (1.32, 1.68) |

Supplementary Table 2: Oral and Pharynx cancer incidence rates and Five-year relative survival rates by census tract Persistent Poverty

|  | **Persistent Poverty** | **Not-Persistent Poverty** |
| --- | --- | --- |
| Age-adjusted Incidence rates (per 100,000)^a^ | | |
| **Sex** |  |  |
| Female | 9.5 (8.9, 10.0)# | 8.9 (8.8, 9.0) |
| Male | 28.5 (27.5. 29.5)# | 24.7 (24.5, 24.9) |
|  |  |  |
| **Race/ethnicity** |  |  |
| NH-White | 18.7 (18.1, 19.2)# | 16.3 (16.2, 16.4) |
| NH-Black | 16.7 (16.1, 17.3)# | 11.2 (10.9, 11.4) |
| NH-Asian/PI | 11.6 (10.5, 12.7) | 10.6 (10.4, 10.9) |
| Hispanic | 8.0 (7.5, 8.5)# | 8.6 (8.4, 8.8) |
|  |  |  |
| **Rurality** |  |  |
| Urban | 14.7 (14.4, 15.1)# | 14.3 (14.2, 14.4) |
| Rural | 14.6 (14.0, 15.2)# | 15.5 (15.2, 15.8) |
|  |  |  |
| Five-year relative survival rates (%, 95% CI) | | |
| **Sex** |  |  |
| Female | 53.7 (51.3, 56.2) | 68.1 (67.4, 68.9) |
| Male | 45.8 (44.3, 47.3) | 66.7 (66.3, 67.2) |
|  |  |  |
| **Race/ethnicity** |  |  |
| NH-White | 53.2 (51.3-55.1) | 68.6 (68.2, 69.1) |
| NH-Black | 39.1 (37.0-41.2)* | 52.9 (51.3-54.4) |
| NH-Asian/PI | 50.6 (44.6-56.3)* | 67.9 (66.6-69.2) |
| Hispanic | 52.8 (49.3-56.2) | 64.0 (62.6-65.4) |
|  |  |  |
| **Rurality** |  |  |
| Urban | 46.5 (45.0, 47.9) | 67.4 (67.0, 67.8) |
| Rural | 52.5 (49.9, 49.8) | 65.1 (63.9, 66.3) |

^a^ Incidence rates are per 100,000 and age-adjusted to the 2000 US standard population.

# P value <0.05, indicates that the rate ratio is significantly different than the rate for Non- persistent poverty census tract. *The relative cumulative survival increased from a prior interval.
